# Autumn Migration to Higher Latitudes in Seminole Bats (*Lasiurus seminolus*) Redefines Seasonal Ranges: Evidence From Stable Isotopes and Fatality Data From Wind Energy Facilities

**DOI:** 10.1002/ece3.71657

**Published:** 2025-06-26

**Authors:** Julia R. Wilson, Michael C. True, Caitlin J. Campbell

**Affiliations:** ^1^ Western EcoSystems Technology, Inc. (WEST) Cheyenne Wyoming USA; ^2^ SWCA Environmental Consultants, Inc. Lombard Illinois USA; ^3^ Bat Conservation International Austin Texas USA; ^4^ Department of Biology University of Florida Gainesville Florida USA

**Keywords:** annual cycle, migratory bat, novel movement, phenology, range extent, renewable energy, seasonal movement

## Abstract

The linkages between individual migrations and population‐level distributions are essential for understanding ecological dynamics. Here we describe how an unconventional migratory strategy in the Seminole bat ( *Lasiurus seminolus* ) results in an extended autumn range, both north and west of its currently described range. Awareness of this redefined distribution will likely reduce possible misidentification with a similar species, the eastern red bat ( *L. borealis* ), during any form of monitoring for purposes of conservation and management efforts. At wind energy facilities during the autumn seasons of 2019–2022 in Iowa, Illinois, Indiana, Michigan, Ohio, and Maryland, we discovered 49 Seminole bat carcasses as fatalities in autumn. All were outside of the species' currently described range in the southeastern United States. For 10 carcasses, we confirmed species identification using molecular techniques. We then modeled the geographic origins of viable fur samples from 17 carcasses using stable hydrogen isotope analysis. Finally, we synthesized current and historical records of the species to redefine seasonal range boundaries. Genetic analysis confirmed that carcasses were Seminole bats, and stable isotope analysis revealed that all bats sampled had migrated north between summer and autumn, leaving the previously defined species range. Our seasonally explicit range delineation for Seminole bats shows that this species expands its range northward in summer and again expands its range in autumn to the north and west to a region that is 1.3 million km^2^ larger than previously recognized. Northward migration in autumn, and the corresponding range extension, were previously undetected in Seminole bats. Our findings also demonstrate that this species, like its congeners, is susceptible to wind turbine fatality. Although the breadth of conservation risk is unknown, this study highlights the importance of obtaining an accurate understanding of migratory movements and the distribution of species to aid in species identification, management, and conservation.

## Graphical abstract

We found 49 Seminole bats ( *Lasiurus seminolus* ) hundreds of kilometers outside of their currently accepted range boundary in autumn. Using a combination of genetic and molecular techniques, we determined that individuals had migrated north from the interior of their core range to well outside of the range. The abundance and consistency in these observations support the redefinition of seasonal range boundaries.

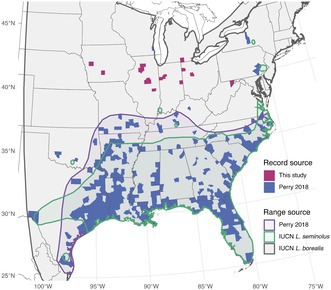

## 1 Introduction

Animal migration is a fundamental ecological phenomenon with marked effects on the structure and functions of ecosystems worldwide (Bauer and Hoye 2014). Accurate descriptions of the seasonal movements of organisms are crucial for understanding basic ecological dynamics, which is foundational for effective conservation and management (Faaborg et al. 2010; La Sorte et al. 2015; Marra et al. 2015). The field of movement ecology has developed a great body of research and technologies, providing a wide array of conservation actions and tools to managers across the globe (Flack et al. 2022; Fudickar et al. 2021). However, migration remains under‐studied in some species groups that are not easily tracked or observed incidentally, such as bats (Robinson et al. 2009; Kays et al. 2015). Recent advancements in stable isotope analyses and tracking technologies have revealed unique and unexpected migratory patterns that have challenged previously held beliefs for these cryptic species (Popa‐Lisseanu and Voigt 2009; Krauel and McCracken 2013; Cryan et al. 2014; Lehnert et al. 2018).

**FIGURE 1:**
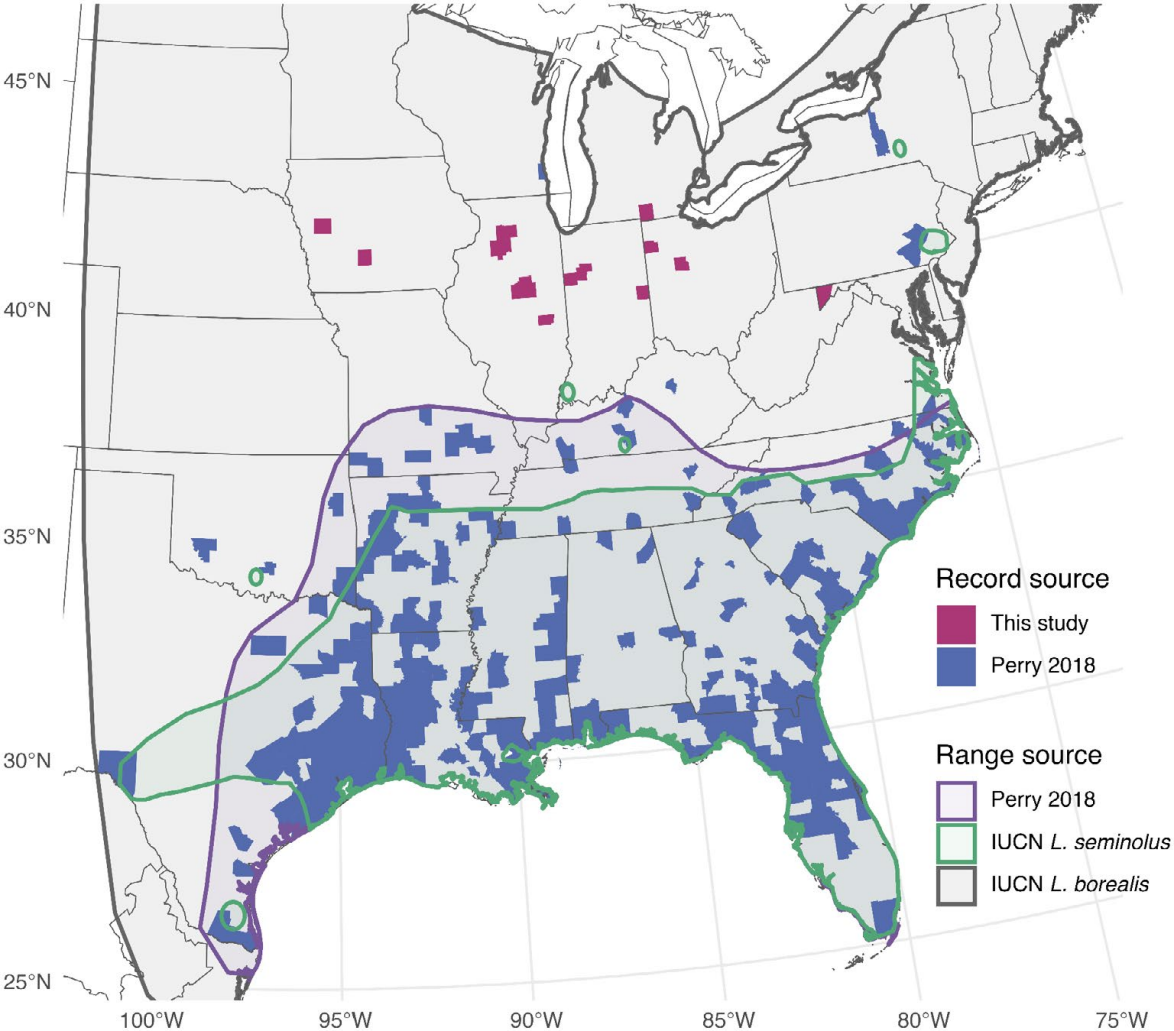
County‐level occurrence records of Seminole bats ( *Lasiurus seminolus* ) from the literature and this study are overlaid with the International Union for Conservation of Nature (IUCN) range map and summer‐occupied range maps from Perry (2018). Map projection is Albers.

In North America, the long‐distance migratory hoary ( *Lasiurus cinereus* ) and eastern red bats ( *L. borealis* ) were originally thought to engage in generally southern migration in autumn, although this expectation was not confirmed by subsequent analyses (Cryan et al. 2014). However, recent stable isotope analyses revealed northward autumnal movements in many individuals (Campbell et al. 2024) and tracking of a small number of hoary bats revealed an autumn “wandering” behavior (Weller et al. 2016; Morningstar and Sandilands 2019). Travel to higher latitudes in autumn has also been documented in tricolored bats ( *Perimyotis subflavus* ; Smith et al. 2022) and Nathusius' pipistrelle bats ( *Pipistrellus nathusii* ; Voigt et al. 2023). Although hoary and eastern red bats engage in unique long‐distance migratory strategies, the closely‐related Seminole bats ( *L. seminolus* ) were not thought to migrate at all until recently (Perry 2018). The Seminole bat is a tree‐roosting red bat (subgenus *Lasiurus*) found throughout the southeastern United States (Barkalow 1948; Barbour and Davis 1969; Wilkins 1987). They are similar in appearance and acoustic vocalization structure to the wider‐ranging eastern red bat, which co‐occur throughout the Seminole bat's range (Figure 1). Seminole and eastern red bats are distinguished by the former having a darker mahogany‐colored pelage (Barbour and Davis 1969). The Seminole bat also occupies similar habitats to the eastern red bat, but with an apparent preference for roosting in pines (*Pinus* spp.; Perry 2018; Barbour and Davis 1969; Hein et al. 2005).

Although Seminole bats were not generally thought to be migratory (Barkalow 1948), extralimital records of Seminole bats in New York, Pennsylvania, and Wisconsin suggest that the species is capable of long‐distance movement (Wilkins 1987; Perry 2018). These records have historically been discounted as outliers representing bats that have been “blown off course” by autumn storms (Wilkins 1987; Perry 2018). A recent review of Seminole bat records defined seasonal ranges, revealing an apparent short‐distance seasonal migratory behavior (Perry 2018). The study also revealed an apparent northward range expansion of 521 km since 1970, with the leading hypothesis of the range expansion being induced by climate change (Wilhide et al. 1998; Lacki 2015; Perry 2018; True et al. 2021). A possible range expansion, autumn movements to higher latitudes, or both, could provide Seminole bats with additional habitat beyond their known range, but also could expose them to other novel stressors.

Autumn migration of hoary and eastern red bats coincides with high mortality rates at wind energy facilities across these species' ranges (Cryan and Brown 2007; Kunz et al. 2007; Arnett and Baerwald 2013; Thompson et al. 2017; Campbell et al. 2024). However, there are currently low levels of wind energy production within the known Seminole bat range (Hoen et al. 2018), and the potential impacts on the species have been assumed to be negligible. Of bats found as fatalities at wind energy facilities across the United States, Seminole bats are estimated to comprise less than 0.1% (American Wind Wildlife Institute [AWWI] 2020). Considering the recent discoveries of cryptic migration strategies in closely‐related species, as well as a possible Seminole bat migration proposed by Perry (2018), accurately describing the seasonal migratory behaviors and geographic range of the Seminole bat is increasingly important. It is possible that Seminole bats have shared seasonal behavioral traits with other Lasiurine species that may increase the risk of fatality at wind turbines in the eastern United States.

During standard post‐construction mortality monitoring (PCMM) of avian and bat fatalities at wind energy facilities, we discovered carcasses of suspected Seminole bats outside of their previously described range in autumn 2019–2022. Our first objective was to assess whether the probable summer origins of these Seminole bats were within the known range of the species and identify migration direction and minimum distance traveled. Our next objective was to use historical and contemporary records (Perry 2018) to identify the season‐specific ranges of the Seminole bat and identify the degree of overlap with wind energy development in the eastern United States. Our results have important implications for the conservation and management of Seminole bats and highlight the need for describing accurate seasonal species range extents that consider complex migratory strategies.

## 2 Methods

### 2.1 Sample Acquisition and Species Identification

We extracted Seminole bat carcass observations from the Western EcoSystems Technology Inc (WEST) database of PCMM studies conducted at wind energy facilities in the Great Plains, Midwest, and eastern United States (limiting the search to North Dakota, South Dakota, Nebraska, Kansas, Oklahoma, Texas, and states east of these) from 2019 to 2022. In the PCMM studies, technicians or detection‐dog teams searched for bird and bat fatalities beneath wind turbines on plots of predetermined sizes at regular search intervals ranging from once per day to once per month. Bias trials were also conducted to account for carcasses that were missed by searchers and carcasses that were unable to be found due to scavenging and decomposition. Plot sizes and search intervals varied between studies depending on PCMM requirements and rotor diameter of turbines. Accordingly, the level of search effort and efficiency varied between studies, resulting in varied detection probabilities of carcasses. The date range of studies also varied, with some occurring year‐round and some occurring only over part of the year (roughly August 1–November 1, during which most bat fatalities occur in North America).

As standard practice, all bat carcasses were photographed, collected, and stored in freezers at a facility onsite until the conclusion of the PCMM study. Trained biologists reviewed each carcass photograph. When a biologist encountered photographs of potential Seminole bats, these photographs were flagged for in‐person evaluation. At the conclusion of the PCMM studies, all bat carcasses were assessed in‐hand and identified to species if possible. In‐hand evaluation of potential Seminole bats included the careful consideration of pelage color for dark mahogany‐brown fur. Eastern red bats and Seminole bats strongly resemble one another, with pelage color being the only differentially diagnostic feature (Wilkins 1987). Thus, the two species can be challenging to distinguish and are presumably prone to misidentification, especially in areas where both species are not expected to occur (i.e., outside of the typical range of the Seminole bat [Perry 2018]) and if found as partially decomposed or scavenged carcasses (Chipps et al. 2020; see also Nelson et al. 2018). To verify accuracy of in‐hand Seminole bat identifications, we collected skin tissue (~25 mm^2^, as in Korstian et al. 2013) from the plagiopatagium or the uropatagium (seven carcasses) or fur from the scapular region (three) from a subset of Seminole bat carcasses (representing up to two carcasses per county) for DNA‐based species identification (for all samples), and to opportunistically determine chromosomal sex (for tissue samples only). These samples were analyzed using qPCR at the Dr. Jane Huffman Wildlife Genetics Institute (East Stroudsburg University, Pennsylvania). Species identification was performed with a sequence targeting the cytochrome *b* (cyt*b*) gene, analyzed using the National Centers for Biotechnology Information database (as in Korstian et al. 2015). Sex was identified using the zinc finder Y‐chromosomal protein gene to target the Y chromosome, and amplification was visualized using gel electrophoresis (as in Korstian et al. 2013; primers from Clare et al. 2007; Ivanova et al. 2007).

### 2.2 Stable Hydrogen Isotope Analysis of Origins

We conducted stable hydrogen isotope analysis of Seminole bat fur, which is expected to be molted in the summer (Pylant et al. 2014) and reflects the isotopic composition of the local precipitation where the fur was formed. Fur samples were collected from all available Seminole bat carcasses from 2020 (19 carcasses; of which 10 were included in DNA methods) by trimming a small amount (~1 mg) of fur from between the scapulae. Fur sample preparation and stable hydrogen isotope analysis was conducted at the University of Maryland Central Appalachians Stable Isotope Facility (CASIF; as in Pylant et al. 2014; Campbell et al. 2020). In brief, samples were washed in 1:200 Triton‐X detergent, rinsed in deionized water, and rinsed in 100% ethanol before air drying (Coplen and Qi 2012). To account for any exchangeable hydrogen remaining in samples from ambient vapor, we applied comparative equilibration in which samples were equilibrated and analyzed concurrently with respect to standards representing known values of non‐exchangeable hydrogen. Approximately 0.15 mg of sample and standard were exposed to ambient air for at least 72 h prior to analysis. Standards applied were four international standards (USGS42, −72.9‰; USGS43, 44.4‰; CBS [Caribou Hoof Standard], −157.0; and KHS [Kudu Horn Standard], −35.3‰; Wassenaar and Hobson 2010; Coplen and Qi 2012) and an internal keratin standard (porcine hair and skin, product # K3030; Spectrum Chemicals, New Brunswick, New Jersey, USA). Analysis for stable hydrogen isotope values (*δ* ^2^H) was conducted with a ThermoFisher high temperature conversion/elemental analyzer pyrolysis unit interfaced with a ThermoFisher Delta V+ isotope ratio mass spectrometer. Values of *δ* ^2^H for bat fur (*δ* ^2^H_fur_) are reported in parts per mille (‰) with reference to the Vienna Standard Mean Ocean Water‐Standard Light Antarctic Precipitation scale. Reported sample precision for this laboratory is 2.3‰ for 𝛿^2^H.

To generate probability‐of‐origin maps for each fur sample, we relied on the published relationship between *δ* ^2^H of precipitation (*δ* ^2^H_precip_) and eastern red bat fur (*δ* ^2^H_fur_; the closest extant relative of Seminole bats) from Campbell et al. (2024). Physiological and life‐history characteristics between the two species are sufficiently similar to expect the same relationship between *δ* ^2^H_precip_ and *δ* ^2^H_fur_ among members of both species, and we note that thoughtfully selected rescaling functions are commonly applied across taxonomic groups (Vander Zanden et al. 2018). This rescaling function was fit with respect to *δ* ^2^H_precip_ values from a mean annual precipitation model that we sourced in approximately 3 km resolution from Bowen et al. (2005) and Bowen and Revenaugh (2003) and reprojected to an Albers equal‐area projection centered on eastern North America. We constrained the potential origins of each fur sample to within a 250‐km buffer around the known range of the Seminole bat that we mapped by creating a 100‐km alpha hull around the centroids of counties with published Seminole bat occurrence records (Perry 2018). We generated probability‐of‐origin maps for each fur sample using the ‘*isotopeAssignmentModel’* function of the R package ‘*isocat*’ (v. 0.2.6; Campbell 2020; Campbell et al. 2020).

We extracted two key metrics summarizing each probability‐of‐origin map: direction of summer origin and minimum distance of travel from that origin. We estimated the most likely direction of fur sample origin relative to the sample site of each bat. To do so, we sampled potential fur origins over 10,000 bootstrap replications weighted by the probability‐of‐origin at each site. For each replication, we calculated the bearing of origin relative to the sample site following the shortest path on an ellipsoid. We categorized samples as having a high probability‐of‐origin to the north or south of the sample site if over 75% of the replications indicated absolute bearings of over or under 90°, respectively. To estimate minimum distance of travel, we defined a “likely” region of origin using the quantile‐simulation transformation described in Campbell et al. (2024). This transformation incorporates model performance on a test dataset of known‐origin samples to achieve correspondence between transformed probability and model accuracy on the test dataset. We incorporated a test dataset of eastern red bat fur samples collected during the documented period between molt and any autumn movement (June 14–August 7; Pylant et al. 2014) sourced from Campbell et al. (2024). We set the probability threshold above which a potential origin would be considered “likely” at one corresponding to 75% accurate assignments for the test dataset. We then quantified the minimum distance of travel for each carcass as the shortest distance on an ellipsoid connecting the sample site to the nearest likely origin of its fur sample. To visualize the likely region of origins of all samples analyzed for stable hydrogen isotope composition, we mapped the cumulative proportion of all likely origins of all sampled bats via overlaying each likely origin map.

All analyses were conducted in R version 4.1.2 and relied heavily on the ‘*isocat*’, ‘*geosphere*’, ‘*sf*’, ‘*raster*’, and ‘*tidyverse*’ packages (Pebesma 2018; Hijmans 2019, 2020; Wickham et al. 2019; Campbell 2020).

### 2.3 Seasonal Range Delineation

We aggregated records identified in this study with those collated previously by Perry (2018). All records were identified to the county‐level. We aggregated records using the seasons defined in Perry (2018), with spring and summer identified as March 16–August 5, autumn as August 6–November 15, and winter as November 16–March 15. We then generated seasonally explicit ranges for Seminole bats using dynamic alpha hulls, which is a common approach for generating extent‐of‐occurrence maps (R package ‘*rangebuilder*’ v.1.6; Davis Rabosky et al. 2016). For each season, we generated dynamic alpha hulls that included at minimum 95% of occurrences within a single hull continuous, constrained to terrestrial land area. We specified a 50 km buffer around points to be included in each hull. We defined “year‐round” range as the overlap between summer and winter ranges, and calculated season‐only ranges as the non‐overlapping area between year‐round and summer, winter, and autumn ranges.

## 3 Results

### 3.1 Sample Acquisition and Species Identification

We identified 49 Seminole bat carcasses during 19 PCMM studies at 13 wind energy facilities outside of the range boundary described in Perry (2018) across 6 states and 13 counties between 2019 and 2022 (Table 1; Figure 1). The subset of 10 samples that were sent for DNA‐based species identification were all verified to be Seminole bats, so we assumed that biologists accurately identified the remaining carcasses that were not confirmed with genetic testing. For carcasses in which skin tissue samples were taken (seven), chromosomal sex determination revealed four carcasses were female and three were male. The remaining three DNA samples were not analyzed genetically for sex identification because only fur was taken, but two of these carcasses were identified based on visual inspection of morphological characteristics (one female, one male). In total, this amounted to five females, four males, and one unknown bat in the subset.

**TABLE 1:** *The number of Seminole bat* ( *Lasiurus seminolus* ) fatalities found during 19 post‐construction mortality monitoring studies at 13 wind energy facilities from 2019 to 2022.

	Iowa	Illinois	Indiana	Michigan	Ohio	Maryland	Total
2019	0	0	7	0	0	0	7
2020	0	3	9	0	7	0	19
2021	0	2	2	4	10	0	18
2022	2	1	1	0	0	1	5
Total	2	6	19	4	17	1	49

Seminole bat carcasses were identified in Iowa, Illinois, Indiana, Michigan, Ohio, and Maryland (ranging from 1 to 19 carcasses; Table 1). Carcasses were found August 2–October 13, with a median discovery date of September 16. We found 12 carcasses in August, 29 in September, and 8 in October. All carcasses were found individually, except for two carcasses found in October in Ohio that were clasped in copulation, providing direct evidence of mating behavior. To our knowledge, the Seminole bats found in Iowa (two) and Michigan (four) represent the first records of the species in these states, and the Seminole bat found in Maryland was the first confidently confirmed record of Seminole bats in the state after the tentative identification by Johnson and Gates (2008).

### 3.2 Stable Hydrogen Isotope Analysis of Origins

**FIGURE 2:**
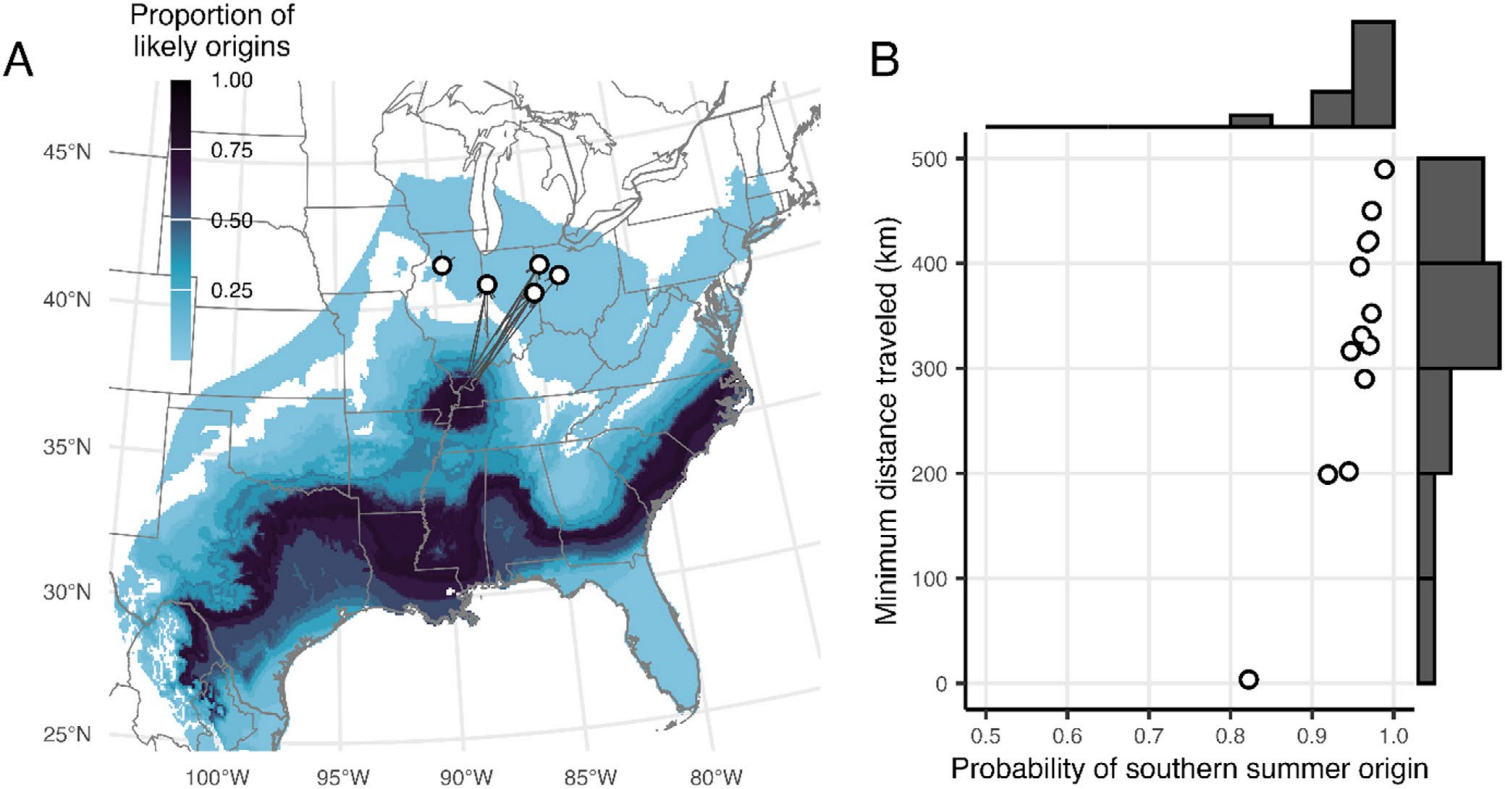
Stable hydrogen isotope analysis of Seminole bat ( *Lasiurus seminolus* ) samples indicated generally southerly summer origins from within the known range, indicating movements in a northern direction outside of the currently described range between summer and autumn (A). The likely origins of the 17 carcasses' fur samples that were analyzed for stable hydrogen isotope values are mapped, where the higher proportion of likely origins is represented by darker color, and where white circles indicate the location where Seminole bats were found and arrows highlight the minimum distance of travel from nearest likely origin. Most samples were associated with a very high likelihood of a summer origin in the southern United States, and the minimum distance traveled for all but one sample was more than 100 km (B). Map projection is Albers.

Of the 19 carcasses with fur samples collected for stable isotope analysis, 17 contained sufficient fur to be successfully analyzed. Those carcasses were found August 5–October 9, 2020, across five wind energy facilities in Illinois (one sample), Indiana (nine), and Ohio (seven; Figure 2A). All reflected a likely southerly origin (87%–98% of replications indicating origins south of the location where the carcass was found; Figure 2B). Most samples reflected likely summer origins that were hundreds of kilometers from the carcass recovery location: 16 samples were associated with minimum distances traveled greater than 100 km, and 8 with travel greater than 400 km (Figure 2B). One sample's likely origin overlapped with the sampling location, and the minimum distance traveled was approximately 5 km, or the resolution of the origin map. All regions of likely origins overlapped with the summer‐occupied range as defined by Perry (2018) (Figure 2A).

### 3.3 Seasonal Range Delineation

**FIGURE 3:**
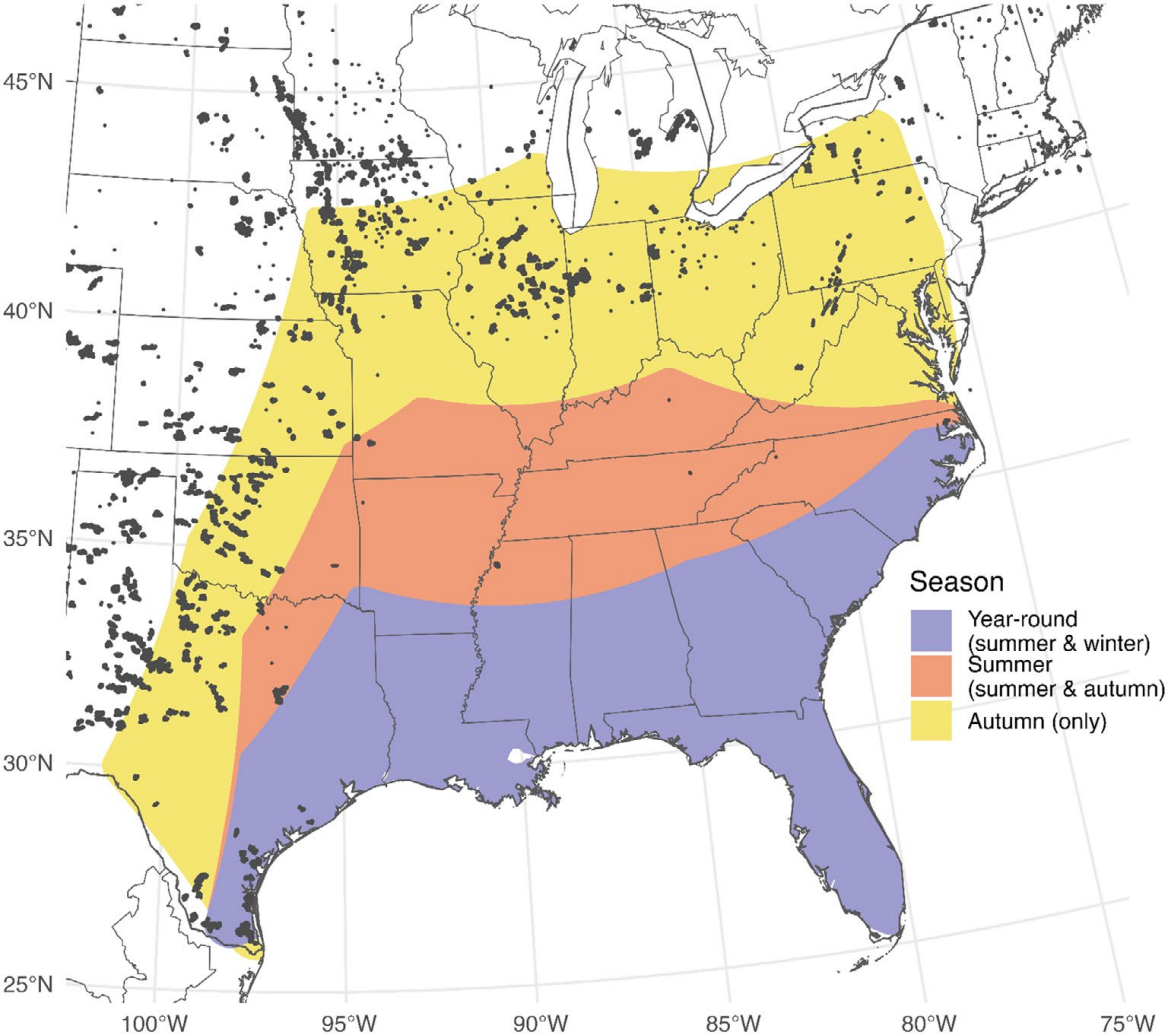
Seasonal ranges of Seminole bats ( *Lasiurus seminolus* ). Year‐round range reflects the overlap of summer and winter ranges; summer range reflects the intersection of summer and autumn ranges, and autumn the range in that season only. Winter range overlaps completely with year‐round range and is not visualized. Gray dots indicate the locations of wind turbines in the United States as of May 2024 (Hoen et al. 2018). Map projection is Albers.

The year‐round range of Seminole bats includes overlap of the winter, summer, and autumn ranges. The area of winter‐only range, not overlapping with the summer range, was < 1 km^2^ and excluded from subsequent analyses and plots (Figure 3). The area occupied in the summer expands upon the winter range by several hundred kilometers in most areas: in the center of the range, that region extends from northern Georgia to northern Kentucky. The autumn range contains and extends further north and west of the summer and year‐round ranges, from central Texas to central Iowa, southern Wisconsin, and upstate New York (Figure 3). The autumn range, where not overlapping with summer or winter range, is 1,334,578 km^2^.

## 4 Discussion

We discovered that some Seminole bats engage in long‐distance migrations to higher latitudes in the autumn, far past the northern boundaries of the species' previously defined range. Such migrations appear to be consistently repeated, considering the abundance of Seminole bats identified in this study over a 4‐year period (Table 1). Distant northern and western autumn records (previously described as “extralimital” or “vagrant”) have been documented for this species since the early 1900s (Poole 1932, 1949; Layne 1955; Lacki 2015; Perry 2018). Given that the stable hydrogen isotope composition of the analyzed samples suggests origins that closely correspond with the summer range as previously described (Perry 2018), the observed northward movement in autumn appears to reflect an explicit seasonal migratory pattern rather than a recent extension of the Seminole bat's summer range.

Here we describe the first direct evidence of long‐distance migrations in Seminole bats: the greatest minimum distance traveled by any Seminole bat in this study was 533 km. Because stable isotope analysis does not provide precise geographic estimates of animal origins, this is necessarily a conservative estimate of distance traveled; the actual route traveled by individuals was likely much longer (e.g., Weller et al. 2016 described a highly circuitous autumn route undertaken by a male hoary bat). We conclude that Seminole bats should be considered likely partial long‐ or mid‐distance migrants (Fleming et al. 2003). Northward autumn movement to higher latitudes has recently been documented in two congeners, the hoary and eastern red bat (Campbell et al. 2024), but was unexpected among Seminole bats, as the species was not previously thought to make long‐distance movements (Barkalow Jr. 1948; Perry 2018). We also note that Seminole bats were previously assumed to be closely associated with Spanish moss ( *Tillandsia usneoides* ) and longleaf pine ( *Pinus palustris* ) stands (Barbour and Davis 1969; Hein et al. 2005), yet our newly defined autumn range does not particularly overlap with pine stands or even with forested habitat. Thus, Seminole bats may not be as restricted by specialized habitat requirements as previously believed, particularly during autumn migration.

We introduce an updated range map that reflects the likely seasonal ranges of the Seminole bat, which is presumably present throughout much of the Midwest and eastern United States in autumn. Given the presence of “extralimital” occurrence records from the 20th and 21st centuries, it appears plausible that this range is not solely a product of a recent year‐round range expansion. It is possible that this larger autumn range would not have been detected previously, given that Seminole and eastern red bats are not acoustically distinguishable and most field studies involving live capture of bats are conducted in the summer. It is also plausible that Seminole bats outside of the known range may have been previously mistakenly identified as eastern red bats, given the species' close resemblance. Chipps et al. (2020) showed that the use of molecular techniques to ascertain the species of carcasses collected at wind turbines increased the accuracy of species' identification and, like Seminole bats, led to the discovery of the presence of two closely related bat species outside their previously known geographic ranges (the western red bat [ *Lasiurus blossevillii* ] and western yellow bat [*Dasypterus xanthinus*]). For future post‐construction monitoring at wind energy facilities, we recommend trained biologists be familiar with the updated range extents of eastern red bats and Seminole bats, conduct in‐hand review of bat carcasses, and use molecular techniques for species identification when able.

The observed northward autumn migration aligns with the recent discovery of autumn migration to higher (and then lower) latitudes of the hoary bat and eastern red bats described in Campbell et al. (2024). They proposed two hypotheses that might drive initial autumn movements to higher latitudes: mate‐seeking and prey‐seeking. With the mating period in autumn and the wide‐ranging nature of bats, it is hypothesized that autumn migration is necessary to find and engage with mates, which is potentially incentivized by sex‐partitioned summer habitats. It has been proposed that migratory tree bats may also congregate around the tallest structures on the landscape for mating purposes (Cryan and Brown 2007; Cryan and Barclay 2009; Jameson and Willis 2014), and therefore mating is also a theoretical driver of short‐distance bat attractions to wind turbines (Guest et al. 2022; Jonasson et al. 2024). This behavior has not been previously described in Seminole bats, and we present the first record of mating Seminole bats near a wind turbine, which suggests that mating behavior may be related to turbine proximity in Seminole bats. The other hypothesis, prey‐seeking, suggests migratory bats may seek out large seasonal emergences and movements of insect prey (Rydell et al. 2010; Hu et al. 2016; Krauel, Brown, et al. 2018; Krauel, Ratcliffe, et al. 2018). We also consider the possibility that autumnal northward movements might reflect the autumn dispersal of juveniles (Perry 2018), which is not mutually exclusive with the previously described hypotheses. We were unable to age the carcasses collected as part of this study, but autumn movements, including to higher latitudes, by adults have been well‐documented (Cryan et al. 2014; Hayes et al. 2015; Weller et al. 2016; Campbell et al. 2024).

Seminole bats engaging in long‐distance migrations are likely well‐positioned to colonize more northerly habitats in a warming climate (Perry 2018; True et al. 2021). As temperatures increase globally, the temperature zone whereby cold conditions would limit Seminole bat activity is likely expanding northward and therefore the range accessible to summer and autumn migrating Seminole bats may be similarly expanding (Perry 2018; True et al. 2021). With this range expansion, autumn movements of Seminole bats may be considered “pioneering” behavior—extralimital movements during spring or autumn, followed by maternity summer occupancy in subsequent years. For example, range shifts in birds initiated from pioneering were observed from the 1950s to the 1990s (Johnson 1994). In bats, Genoways et al. (2000) emphasized that “vagrants” are integral to the mechanism of range shifting, and McCracken et al. (2018) suggested that autumn extralimital movements of Brazilian free‐tailed bat ( *Tadarida brasiliensis* ) were in some cases followed by potential expansions of the species' breeding range. Indeed, Perry (2018) noted anecdotal accounts of Seminole bats occurring at the northern edge of their range in autumn, followed by maternity use in subsequent years.

Among North American bats, hoary bats and eastern red bats are impacted most by fatalities at wind energy facilities, which has led to analyses and discussion of conservation concerns (Arnett and Baerwald 2013; Frick et al. 2017; AWWI 2020; Friedenberg and Frick 2021). Possibly due to shared behavioral characteristics with these congener species, individual Seminole bats may face a similar fatality risk when near wind turbines. Therefore, migratory movements across the Midwest and eastern United States may result in interactions of Seminole bats with wind energy development that may pose a higher risk of fatality than previously recognized (although Seminole bats represent less than 0.1% of reported bat fatalities in the United States; AWWI 2020). This risk is also historically uncertain because counts could be biased low due to possible misidentifications between eastern red bats and Seminole bats in areas where these species were not thought to co‐occur. In addition, while overall fatality risk may be low due to a lack of overlap between appreciable wind energy development and the year‐round range of Seminole bats, the expansion of wind energy development southward into the core range may lead to increased collisions. With our better understanding of the Seminole bat autumn range extent and the knowledge that Seminole bats are susceptible to wind turbine fatalities, we recommend that future PCMM studies in the eastern United States account for the potential presence of Seminole bats. We recommend careful planning to account for the challenges of distinguishing Seminole bats from eastern red bats to aid in better understanding the general impacts of wind energy development on Lasiurine species and ensure that accurate impact estimates are generated.

Effective wildlife conservation and management are contingent upon accurate species‐level identification, which relies heavily on a thorough understanding of the geographical distribution of a species. Historically, research and data collection efforts have been heavily biased towards studying warm‐season ecologies across most taxa (Marra et al. 2015). Echoing calls for full annual cycle research in ecology (Marra et al. 2015), this study serves as a crucial example of how uncovering a cryptic migratory strategy and corresponding seasonal range can result in dramatic distribution‐level effects with important conservation and management implications. This study makes the case for future bat migration research to specifically examine how autumnal migratory behavior contributes to a species' seasonal distribution, particularly in the context of global environmental change and interactions with anthropogenic development.

## Author Contributions

**Julia R. Wilson:** conceptualization (lead), data curation (lead), funding acquisition (lead), investigation (equal), methodology (equal), project administration (equal), resources (equal), writing – original draft (equal), writing – review and editing (equal). **Michael C. True:** data curation (equal), formal analysis (equal), funding acquisition (supporting), investigation (equal), methodology (equal), project administration (equal), resources (equal), validation (equal), visualization (equal), writing – original draft (equal), writing – review and editing (equal). **Caitlin J. Campbell:** data curation (supporting), formal analysis (equal), investigation (equal), methodology (equal), project administration (equal), resources (equal), software (equal), validation (equal), visualization (equal), writing – original draft (equal), writing – review and editing (equal).

## Conflicts of Interest

The data used in this study exist due to financial contract relationships between wind energy facilities and Western EcoSystems Technology Inc. (WEST). In completing the study, it required approval from wind energy facilities to use the data anonymously. However, the financial contracts and approval for inclusion did not influence the methods, results, or overall conclusions of the study. MT and JW, authors of this study, were employed by the study's funder, WEST, during the study's conception. Both received standard compensation, including an annual salary and benefits consistent with those provided to all employees in similar roles. Their employment by the funder did not influence the methods, results, or overall conclusions of the study.

## Data Availability

Data and code used to conduct the analyses are available at https://doi.org/10.5061/dryad.79cnp5j7j. As part of agreements to use the data, we used county centroids in place of the exact carcass location to maintain the anonymity of the projects and companies associated. This decision had a negligible impact on the overall conclusions of the study given the inferred travel distance of individuals (hundreds of kilometers) relative to average breadth of counties in the study region (tens of kilometers).
